# Myocardial work in chronic kidney disease: insights from the CPH-CKD ECHO Study

**DOI:** 10.1007/s00392-024-02459-6

**Published:** 2024-05-15

**Authors:** Flemming Javier Olsen, Nino Emanuel Landler, Jacob Christensen, Bo Feldt-Rasmussen, Ditte Hansen, Christina Christoffersen, Ellen Linnea Freese Ballegaard, Ida Maria Hjelm Sørensen, Sasha Saurbrey Bjergfelt, Eline Seidelin, Susanne Bro, Tor Biering-Sørensen

**Affiliations:** 1 Cardiovascular Non-Invasive Imaging Research Laboratory, Department of Cardiology, Copenhagen University Hospital – Herlev and Gentofte, Gentofte Hospitalsvej 8, 2900 Hellerup, Denmark; 2 Department of Biomedical Sciences, University of Copenhagen, Copenhagen, Denmark; 3 Department of Nephrology, Copenhagen University Hospital – Rigshospitalet, Copenhagen, Denmark; 4 Department of Clinical Medicine, University of Copenhagen, Copenhagen, Denmark; 5 Department of Nephrology, Copenhagen University Hospital – Herlev and Gentofte, Herlev, Denmark; 6 Department of Clinical Biochemistry, Copenhagen University Hospital – Rigshospitalet, Copenhagen, Denmark; 7 Department of Cardiology, Copenhagen University Hospital – Rigshospitalet, Copenhagen, Denmark; 8 Steno Diabetes Center Copenhagen, Herlev, Denmark

**Keywords:** Myocardial work, Pressure-strain, Kidney disease, Chronic nephropathy

## Abstract

**Background:**

Myocardial work is a novel echocardiographic measure that offers detailed insights into cardiac mechanics. We sought to characterize cardiac function by myocardial work in patients with chronic kidney disease (CKD).

**Methods:**

We prospectively enrolled 757 patients with non-dialysis-dependent CKD and 174 age- and sex-matched controls. Echocardiographic pressure-strain loop analysis was performed to acquire the global work index (GWI). Linear regressions were performed to investigate the association between estimated glomerular filtration rate (eGFR) and urine albumin-creatinine ratio (UACR) to GWI.

**Results:**

Patients with CKD had a mean age of 57 years, 61% were men, and median eGFR was 42 mL/min/1.73 m^2^. Overall, no difference in GWI was observed between patients and controls (1879 vs. 1943 mmHg%, *p* = 0.06). However, a stepwise decline in GWI was observed for controls vs. patients with CKD without left ventricular hypertrophy vs. patients with CKD and left ventricular hypertrophy (GWI, 1943 vs. 1887 vs. 1789 mmHg%; *p* for trend = 0.030). In patients with CKD, eGFR was not associated with GWI by linear regression. However, diabetes modified this association (*p* for interaction = 0.007), such that per 10 mL/min/1.73 m^2^ decrease in eGFR, GWI decreased by 22 (9–35) mmHg% (*p* = 0.001) after multivariable adjustments in patients without diabetes, but with no association between eGFR and GWI in patients with diabetes. No association was observed between UACR and GWI.

**Conclusion:**

Patients with CKD and left ventricular hypertrophy exhibited lower myocardial work compared to matched controls. Furthermore, decreasing eGFR was associated with decreasing myocardial work only in patients without diabetes. No association to UACR was observed.

**Graphical Abstract:**

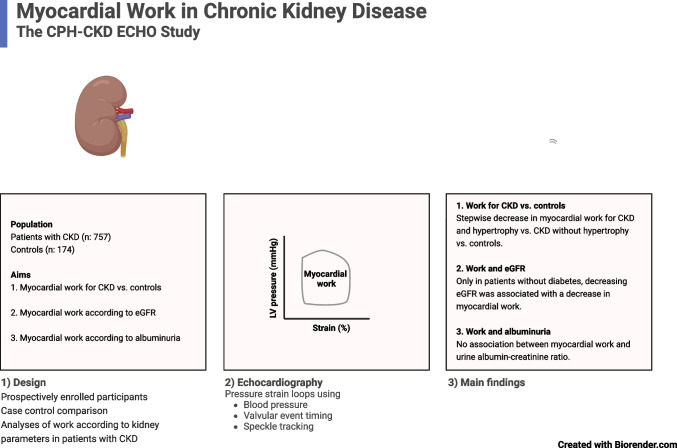

**Supplementary Information:**

The online version contains supplementary material available at 10.1007/s00392-024-02459-6.

## Introduction

Chronic kidney disease (CKD) is increasing worldwide, with an estimated prevalence of 13% [1, 2]. Patients with CKD face an elevated risk of heart failure (HF) and those who develop HF have a high risk of HF readmissions, CKD progression, and all-cause death [3–5]. This emphasizes the importance of timely recognition of patients at risk of HF who may benefit from close monitoring to guide preventive measures. Transthoracic echocardiography could be valuable in this regard as it is a cost-effective, harmless, and readily available tool in the clinic. Although cardiac abnormalities are frequently observed with echocardiography in patients with CKD, systolic dysfunction is overall not a common finding with quite diverging prevalence estimates of 2–18% [6–8].

This may reflect that systolic dysfunction has conventionally been defined from the left ventricular ejection fraction (LVEF), which has several technical limitations [9]. Consequently, novel techniques have been developed to provide a more detailed evaluation of systolic function. One such technique contemplates the use of pressure-strain loop analyses as a non-invasive method to estimate myocardial work [10]. This method provides a direct assessment of myocardial tissue function in a highly reproducible manner while also taking afterload into consideration, thus potentially superior to global longitudinal strain (GLS) [11]. This method may therefore provide a more nuanced characterization of myocardial contraction in patients with CKD as a means to identify patients at risk of HF. Consequently, in this study, we sought to provide a detailed characterization of systolic function. We hypothesized that patients with CKD exhibited more abnormal myocardial work compared to controls and that myocardial work measures worsened with decreasing renal function.

## Methods

### Population

We performed a cross-sectional analysis based on participants who were prospectively enrolled in the Copenhagen Chronic Kidney Disease Echocardiographic (CPH-CKD ECHO) Study. The study has previously been described in detail [12, 13]. Briefly, the CPH-CKD ECHO Study was a dual-centre study that comprised patients with non-dialysis-dependent CKD (*n*, 825) and age- and sex-matched control individuals (*n*, 175). Patients with CKD were enrolled from the outpatient clinic at the Departments of Nephrology at Rigshospitalet and Herlev-Gentofte Hospital in Denmark. Controls were recruited through posts in local newspapers and on a Danish website (forsoegsperson.dk) dedicated to highlighting ongoing research studies seeking voluntary individuals. The inclusion phase ran from October 2015 through October 2018. Inclusion criteria were known CKD and age between 30 and 75 years. CKD was defined as either kidney injury (albuminuria, renal cyst, or other structural abnormalities in the kidneys) or an eGFR < 60 mL/min/1.73 m^2^ lasting more than 3 months [14]. Exclusion criteria were as follows: kidney transplantation with a functioning graft, pregnancy, intellectual disability, dementia or psychosis, active malignancy, and retraction of informed consent.

The controls were not allowed to have known cardiovascular disease, kidney disease, malignant disease, or chronic disease, apart from mild hypertension, thyroid disease, mild depression, or hypercholesterolemia. Subjects were excluded if they had kidney damage or an eGFR < 60 mL/min/1.73 m^2^.

The total study population thus included 1000 individuals. Of these, 9 were excluded because they had aortic valve stenosis since the myocardial work analysis assumes that there is no obstruction between the left ventricle and the aorta [10]. In addition, 5 were excluded because they had severe aortic regurgitation since myocardial work analysis assumes negligible diastolic pressures, which is not the case with severe aortic regurgitation [10]. Finally, 55 were excluded because the myocardial work analysis was not technically feasible, leaving 931 for final analysis (757 patients with CKD and 174 controls).

### Ethics

The study was approved by the regional scientific ethics committee (ID: H-3–2011-069) and the Danish Data Protection Agency (ID: 30–0840). Informed consent was obtained from all participants, and the study adhered to the 2^nd^ Helsinki Declaration.

### Clinical characteristics

Data on medical history and clinical characteristics were collected at an outpatient baseline visit upon inclusion. Medical history was obtained by interview and review of hospital medical records. A physical examination was performed to measure anthropometrics, heart rate, and brachial artery blood pressure. Hypertension was defined as a systolic blood pressure > 140 mmHg, diastolic blood pressure > 90 mmHg, or the use of antihypertensive medication. Urinary samples were collected to determine the degree of albuminuria (urine albumin-creatinine ratio, UACR). Details on UACR were available in 733/757 patients. Venous blood samples were drawn to acquire plasma creatinine level and for storage in a biobank. A 12-lead electrocardiogram was also performed at the visit.

eGFR was calculated from plasma creatinine level with the CKD-EPI formula [15]. The patients were grouped into eGFR categories in accordance with the 2012 Kidney Disease Improving Global Outcomes (KDIGO) guidelines [14]. In line with our previous publications, we also collated patients into three eGFR groups as follows: G1 + G2 (eGFR ≥ 60 mL/min/1.73 m^2^), G3 (eGFR 30–59 mL/min/1.73 m^2^), and G4 + G5 (eGFR ≤ 29 mL/min/1.73 m^2^) [13].

Albuminuria was characterized as follows: normoalbuminuria with UACR < 30 mg/g, microalbuminuria with UACR of 30–300 mg/g, and macroalbuminuria with UACR > 300 mg/g.

### Standard echocardiography

All echocardiographic examinations were performed using a GE Vivid E9 ultrasound machine according to a dedicated protocol. All images were acquired over three consecutive cycles. For individuals in sinus rhythm, measurements were performed in a single cardiac cycle — the one with the most optimal image quality — whereas all measurements were performed in three cardiac cycles for patients who had atrial fibrillation during the examination. All standard measures were performed with commercially available post-processing software (EchoPAC BT 203, GE Healthcare) by a single experienced investigator blinded to clinical information according to the 2015 American Society of Echocardiography/European Association of Cardiovascular Imaging (ASE/EACVI) guidelines [16]. The analysis process has been described meticulously elsewhere [13].

Valve disease was quantified according to the most recent guidelines [17, 18]. Significant valve disease was defined as either moderate aortic valve regurgitation, moderate or severe mitral regurgitation, or moderate or severe mitral stenosis.

### Pressure-strain loop analysis

**Fig. 1:**
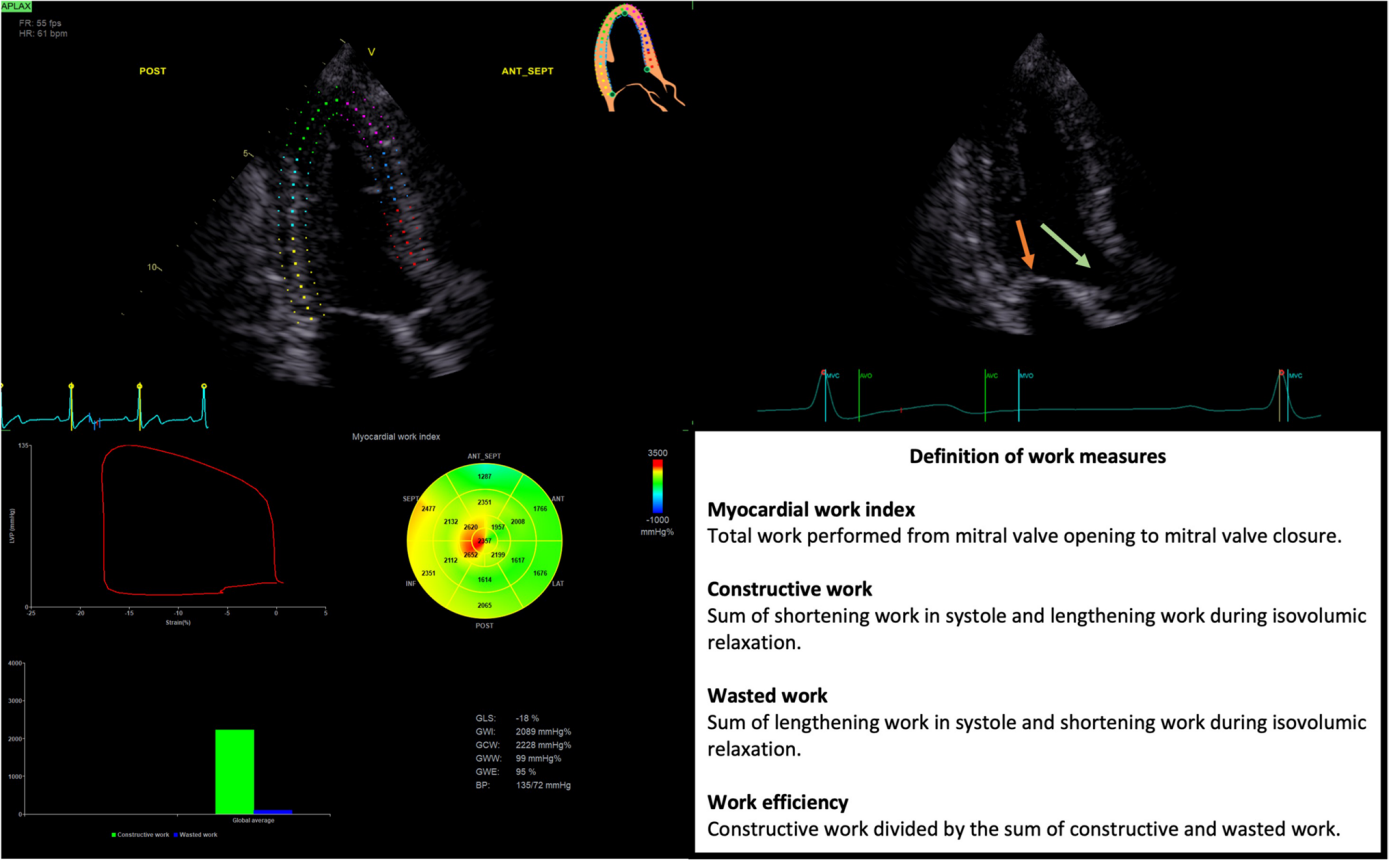
The pressure-strain loop analysis process used to obtain myocardial work measures. First, left ventricular speckle tracking is performed (top left panel), then the brachial artery blood pressure is added, and then the timing of valvular opening and closure is visually estimated (top right panel with orange arrow at the mitral valve and green arrow at the aortic valve). The results are depicted in the bottom left panel and include a pressure-strain loop with the area reflecting global myocardial work index, the bulls-eye plot shows segmental values of myocardial work, and bar charts show the relative distribution of constructive and wasted work. The bottom right panel presents definitions of the four work measures derived from the pressure-strain loop analysis

Analyses of pressure-strain loops were performed according to published directions [19]. The analysis process and definition of work parameters are shown in Fig. 1. Pressure-strain loops were acquired by first analyzing myocardial speckle tracking in the three apical projections (minimum frame rate of 40 frames per second, mean ± SD, 58 ± 5 frames per second). The left ventricular myocardium was automatically traced using automated function imaging. This created a region of interest that covered the endocardial throughout the myo-epicardial layer. The tracing and region of interest could be adjusted at the discretion of the investigator. Segments could be excluded if the tracing did not follow the myocardial speckles adequately, however, only one segment in total could be excluded, otherwise, the analysis was considered infeasible. Speckle tracking analysis was feasible in 941 (94%). Pressure-strain loops were then created by inputting blood pressure and visually estimating valvular event timing. The following myocardial work parameters were derived from the pressure-strain loop analyses: global work index (GWI), global work efficiency (GWE), global constructive work (GCW), and global wasted work (GWW).

Abnormal work indices were defined as follows according to published reference material [20]: GWI < 1576 mmHg%, GCW < 1708 mmHg%, GWW > 159 mmHg%, GWE < 93.0%.

In line with our previous publication, GLS below 18% (numerical value) was considered abnormal [13].

### Statistics

Clinical and echocardiographic characteristics were compared for the patients with CKD stratified by normal vs. abnormal GWI. Furthermore, myocardial work measures were compared between patients with CKD and controls. For these comparisons, Gaussian-distributed continuous variables were analyzed with Student’s *T*-test and reported as mean with standard deviation. Non-Gaussian distributed variables were compared with the Wilcoxon rank-sum test and reported as median with interquartile ranges. Gaussian distribution was assessed from histograms. Categorical variables were compared with either the chi^2^ test or Fisher’s exact test as appropriate and reported as total numbers with percentages.

Linear multivariable regressions were made to account for confounders between patients with CKD and controls and calculate predicted means. Adjustments were made for body mass index, diabetes, hypertension, smoking status, alcohol consumption, eGFR, UACR, heart rate, left bundle branch block, atrial fibrillation, heart failure, ischemic heart disease, significant valve disease, and left ventricular mass index. For all regression analyses involving work measures, the GWW and GWE variables were log- and logit-transformed, respectively.

Comparisons were further made across groups of left ventricular remodeling (controls vs. patients with CKD without and with left ventricular hypertrophy (LVH), respectively), across eGFR groups (G1, G2, G3, G4, G5), strata of albuminuria (normoalbuminuria, microalbuminuria, and macroalbuminuria). For these comparisons, the ANOVA test was applied for Gaussian-distributed variables, and non-Gaussian distributed variables were compared with a non-parametric trend test.

Linear regression analysis was also applied to examine the association between eGFR and myocardial work measures and between UACR and myocardial work measures. For all regression analyses, UACR underwent a log-transformation. Multivariable adjustments were made for relevant confounders: age, sex, hypertension, diabetes, heart rate, significant valve disease, ischemic heart disease, smoking status, alcohol consumption, body mass index, known heart failure, and left ventricular mass index. For the analyses concerning the association between UACR and work measures, the multivariable model also included adjustment for eGFR and vice versa. The same analyses were carried out in a subgroup of patients with CKD who exhibited current signs of functional kidney disease (defined as either reduced eGFR (< 60 mL/min/1.73 m^2^) or albuminuria (UACR > 30 mg/g)).

Logistic regression was applied to investigate which stages of CKD were associated with increased likelihood of abnormal work. Multivariable adjustments were similar to the linear regressions.

Tests for interactions from diabetes, hypertension, and CKD etiology, respectively, were applied in both linear and logistic regression analysis. Since diabetes significantly modified the association between eGFR and work measures, these regression analyses were stratified by diabetes status.

All statistical analyses were performed using STATA v. 15 SE (StataCorp LP, College Station, TX). *p*-values < 0.05 were considered significant in all analyses.

## Results

Table 1 outlines clinical baseline characteristics for all patients with CKD included in the present analysis. Briefly, these patients had a mean age of 57 years, 61% were of male sex, and the median eGFR was 42 mL/min/1.73 m^2^. Comparisons between controls and patients with CKD in terms of clinical characteristics have previously been published [13]. Per study design, known cardiovascular disease and diabetes were absent in controls, who more frequently were non-smokers and had lower BMI, but similar blood pressure compared with patients with CKD.

**Table 1:** Baseline clinical characteristics for patients with CKD

	Patients with CKD *n*, 757	Normal global work index *n*, 613		Abnormal global work index *n*, 144			*p*-value	
Clinical								
Age, years	57 ± 13	57 ± 13		59 ± 12			0.048	
Male sex, *n* (%)	465 (61)	356 (58)		109 (76)			< 0.001	
Body mass index, kg/m^2^*	28 ± 6	28 ± 6		29 ± 5			0.046	
Systolic blood pressure, mmHg	132 ± 17	133 ± 17		128 ± 17			0.002	
Diastolic blood pressure, mmHg	81 ± 11	81 ± 11		82 ± 11			0.84	
Heart rate, beats/minute	72 ± 13	71 ± 12		77 ± 15			< 0.001	
Left bundle branch block, *n* (%)	10 (1)	4 (1)		6 (4)			< 0.001	
Medical history								
Hypertension, *n* (%)	640 (85)	523 (85)		117 (81)			0.17	
Diabetes mellitus, *n* (%)	143 (19)	103 (17)		40 (28)			0.002	
Peripheral artery disease, *n* (%)	38 (5)	22 (4)		16 (11)			< 0.001	
Prior myocardial infarction, *n* (%)	32 (4)	20 (3)		12 (8)			0.007	
Prior coronary artery bypass graft, *n* (%)	26 (3)	16 (3)		10 (7)			0.010	
Prior percutaneous coronary intervention, *n* (%)	22 (3)	14 (2)		8 (6)			0.035	
Atrial fibrillation	55 (7)	32 (5)		23 (16)			< 0.001	
Atrial fibrillation during echocardiographic exam, *n* (%)	20 (3)	5 (1)		15 (10)			< 0.001	
Heart failure, *n* (%)	60 (8)	29 (5)		31 (22)			< 0.001	
Previous stroke or transient ischemic attack, *n* (%)	60 (8)	44 (7)		16 (11)			0.12	
Smoking status, *n* (%)*							0.019	
Never	302 (40)	257 (42)		45 (32)				
Former	133 (18)	107 (18)		26 (18)				
Active	320 (42)	248 (41)		72 (50)				
Alcohol consumption, drinks/week*	2 [0;7]	2 [0;7]		2 [0;9]			0.50	
CKD etiology							0.024	
Diabetic nephropathy, *n* (%)	71 (9)	54 (9)		17 (12)				
Hypertensive or renal vascular nephropathy, *n* (%)	39 (5)	25 (4)		14 (10)				
Tubulointerstitial nephropathy, *n* (%)	12 (2)	10 (2)		2 (1)				
Glomerulonephritis or vasculitis, *n* (%)	239 (32)	200 (33)		39 (27)				
Polycystic kidney disease, *n* (%)	108 (14)	96 (16)		12 (8)				
Other cause**	108 (14)	85 (14)		23 (16)				
Unknown cause	180 (24)	143 (23)		37 (26)				
Biochemistry								
Estimated glomerular filtration rate, mL/min/1.73 m^2^	42 [28;62]	43 [29;64]		41 [26;52]			0.044	
Urine albumin-creatinine ratio, mg/g*	129 [21;681]	119 [20;692]		186 [26;616]			0.42	
Medication								
ACE-I/ARB, *n* (%)	480 (63)	390 (64)		90 (63)			0.80	
Calcium blocker, *n* (%)	287 (38)	241 (39)		46 (32)			0.10	
Beta-blocker, *n* (%)	226 (30)	172 (28)		54 (38)			0.026	
Aldosterone antagonist, *n* (%)	38 (5)	26 (4)		12 (8)			0.043	
Diuretics, *n* (%)	408 (54)	316 (52)		92 (64)			0.008	
Echocardiography								
Significant valve disease, *n* (%)***	15 (2)	10 (2)		5 (4)			0.15	
Left ventricular internal diameter, cm	4.8 ± 0.6	4.8 ± 0.6		4.9 ± 0.7			0.44	
Left ventricular mass index, g/m^2^	75.0 [62.8;89.1]	73.2 [62.1;87.4]		83.6 [64.8;98.3]			< 0.001	
Left ventricular ejection fraction, %	58.4 ± 7.3	59.9 ± 5.6		52.0 ± 9.7			< 0.001	
Left atrial volume index, mL/m^2^	27.1 ± 8.9	27.3 ± 8.0		26.4 ± 12.2			0.32	
E/e′	7.8 [6.4;9.6]	7.8 [6.4;9.6]		7.9 [6.4;10.1]			0.65	

*CKD*, chronic kidney disease; *ACE-I*, angiotensin-converting enzyme inhibitor; *ARB*, angiotensin receptor blocker

^*^Details were missing for the following variables: body mass index (*n*, 1), smoking history (*n*, 2), alcohol consumption (*n*, 3), and urine albumin-creatinine ratio (*n*, 24)

^**^Refers to a heterogeneous group of rare etiologies, including congenital, hereditary, and toxic causes

^***^Includes moderate aortic regurgitation, moderate or severe mitral regurgitation, and moderate or severe mitral stenosis

Table 1 also outlines baseline characteristics for patients with CKD as stratified by abnormal GWI. In brief, those who had abnormal GWI were slightly older, more frequently men, had lower systolic blood pressure, higher heart rate, and an overall higher proportion of cardiovascular risk factors and disease. In terms of renal function, they exhibited slightly lower eGFR, whereas no significant difference in UACR was noted. In terms of echocardiographic characteristics, those with abnormal GWI exhibited lower ejection fraction and higher left ventricular mass index, but similar diastolic function.

### Myocardial work in chronic kidney disease

When comparing patients with CKD to controls, no differences were noted in GWI (1879 vs. 1943 mmHg%, *p* = 0.06) nor GCW (2193 vs. 2249 mmHg%, *p* = 0.11). However, they did exhibit higher GWW (133 vs. 107 mmHg%, *p* < 0.001) and lower GWE (94.3 vs. 95.2%, *p* < 0.001). These findings were unchanged after multivariable adjustments. However, when stratified according to left ventricular geometry, we observed a stepwise increase in GWW and decrease in GWI and GWE for patients with CKD and hypertrophy vs. patients with CKD without hypertrophy vs. controls (GWI: 1789 vs. 1887 vs. 1943 mmHg%, *p* for trend = 0.030; GWW: 194 vs. 130 vs. 107 mmHg%, *p* for trend < 0.001; GWE: 91.6 vs. 94.5 vs. 95.2%, *p* for trend < 0.001) with no differences in GCW (*p* for trend = 0.20). These differences persisted after multivariable adjustments.

**Fig. 2:**
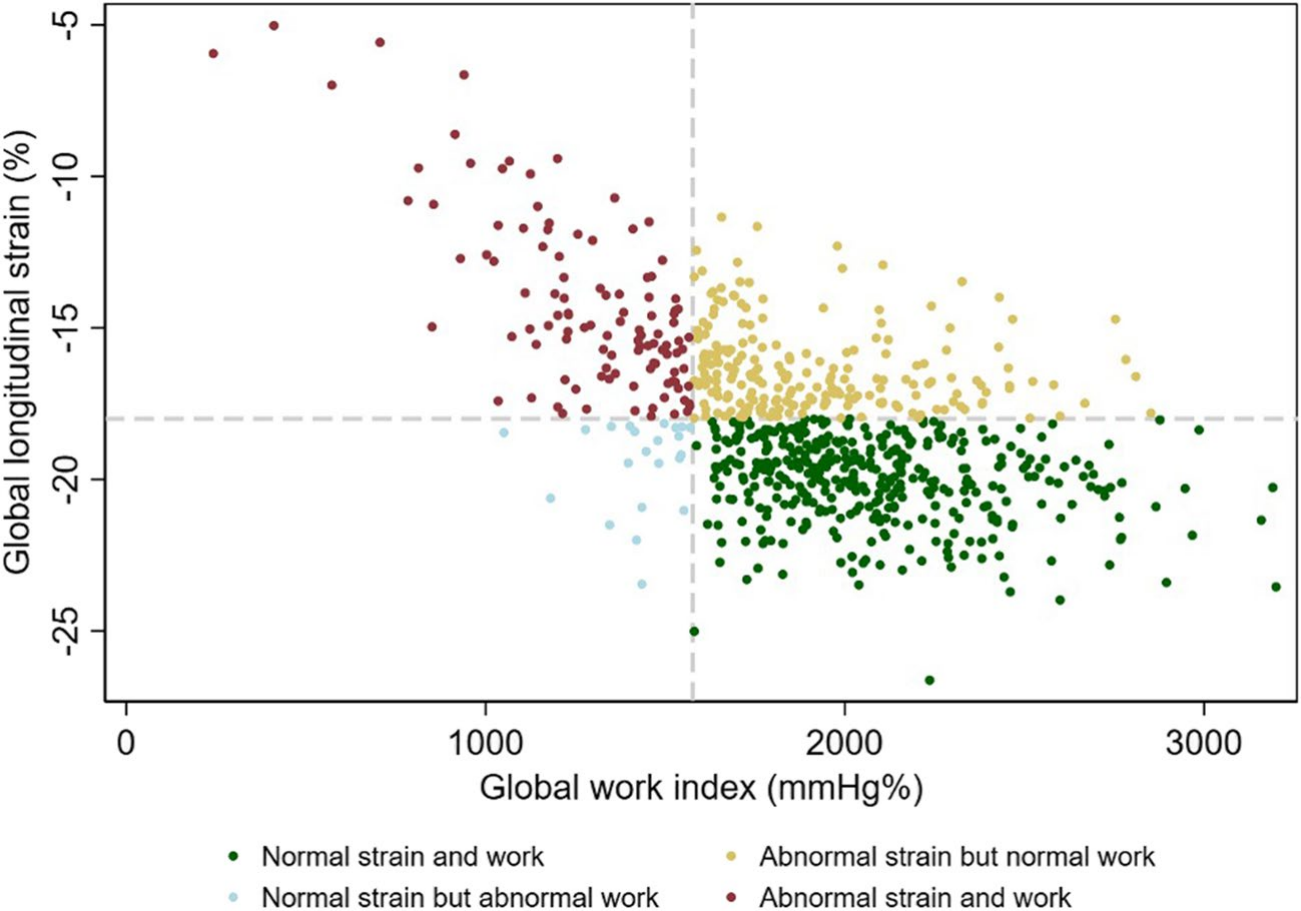
Distribution of myocardial work and longitudinal strain. Separated scatter plot illustrating how measures of global longitudinal strain and myocardial work were distributed according to abnormalities in strain and work

Among patients with CKD, 325 (45%) were considered to have abnormal GLS, whereas 144 (19%) were considered to have abnormal GWI. Accordingly, 216 (66%) of those with abnormal GLS had normal myocardial work. The distribution of values for GLS and GWI according to groups of abnormal strain and work is shown in Fig. 2.

### Myocardial work in relation to eGFR

Table 2 shows absolute values for myocardial work measures as well as the proportion of abnormal work measures according to eGFR groups. A significant trend of higher GWW and lower GWE was observed across the groups. By extension, the proportion of patients with abnormal GWW and GWE increased significantly across the groups. No differences in either GWI or GCW were observed across eGFR groups; however, the proportion of abnormal work indices increased significantly across the groups, being notably higher in the G3, G4, and G5 compared to the G1 and G2 groups.

**Table 2:** Myocardial work according to eGFR groups

	G1 eGFR ≥ 90 mL/min/1.73 m^2^ *n*, 74	G2 eGFR 60–89 mL/min/1.73 m^2^ *n*, 129	G3 eGFR 30–59 mL/min/1.73 m^2^ *n*, 355	G4 eGFR 15–29 mL/min/1.73 m^2^ *n*, 156	G5 eGFR < 15 mL/min/1.73 m^2^ *n*, 43	*p* for trend
Myocardial work measures						
GWI, mmHg%	1911 ± 382	1937 ± 335	1867 ± 420	1853 ± 463	1843 ± 489	0.40
GCW, mmHg%	2184 ± 381	2241 ± 347	2183 ± 434	2191 ± 474	2156 ± 514	0.70
GWW, mmHg%	107 [81;134]	118 [87;162]	141 [103;194]	147 [104;205]	168 [96;242]	< 0.001
GWE, %	95.4 [93.9;96.3]	94.9 [93.2;96.2]	94.1 [91.7;95.6]	94.1 [91.0;95.5]	93.4 [91.1;95.5]	< 0.001
Abnormal work measures						
Abnormal GWI, *n* (%)	10 (14)	11 (9)	79 (22)	34 (22)	10 (23)	0.006
Abnormal GCW, *n* (%)	5 (7)	4 (3)	40 (11)	19 (12)	7 (16)	0.025
Abnormal GWW, *n* (%)	11 (15)	34 (26)	134 (38)	64 (41)	22 (51)	< 0.001
Abnormal GWE, *n* (%)	11 (15)	30 (23)	133 (38)	57 (37)	20 (47)	< 0.001

*eGFR*, estimated glomerular filtration rate; *GWI*, global work index; *GCW*, global constructive work; *GWW*, global wasted work; *GWE*, global work efficiency

Linear regression analysis did not reveal any continuous association between eGFR and either GWI or GCW (*p* = 0.33 and 0.81, respectively) but did show that GWW increased and GWE decreased with decreasing eGFR (*p* < 0.001 for both). However, these associations did not persist after multivariable adjustments (*p* = 0.50 and 0.17 for GWW and GWE, respectively).

**Fig. 3:**
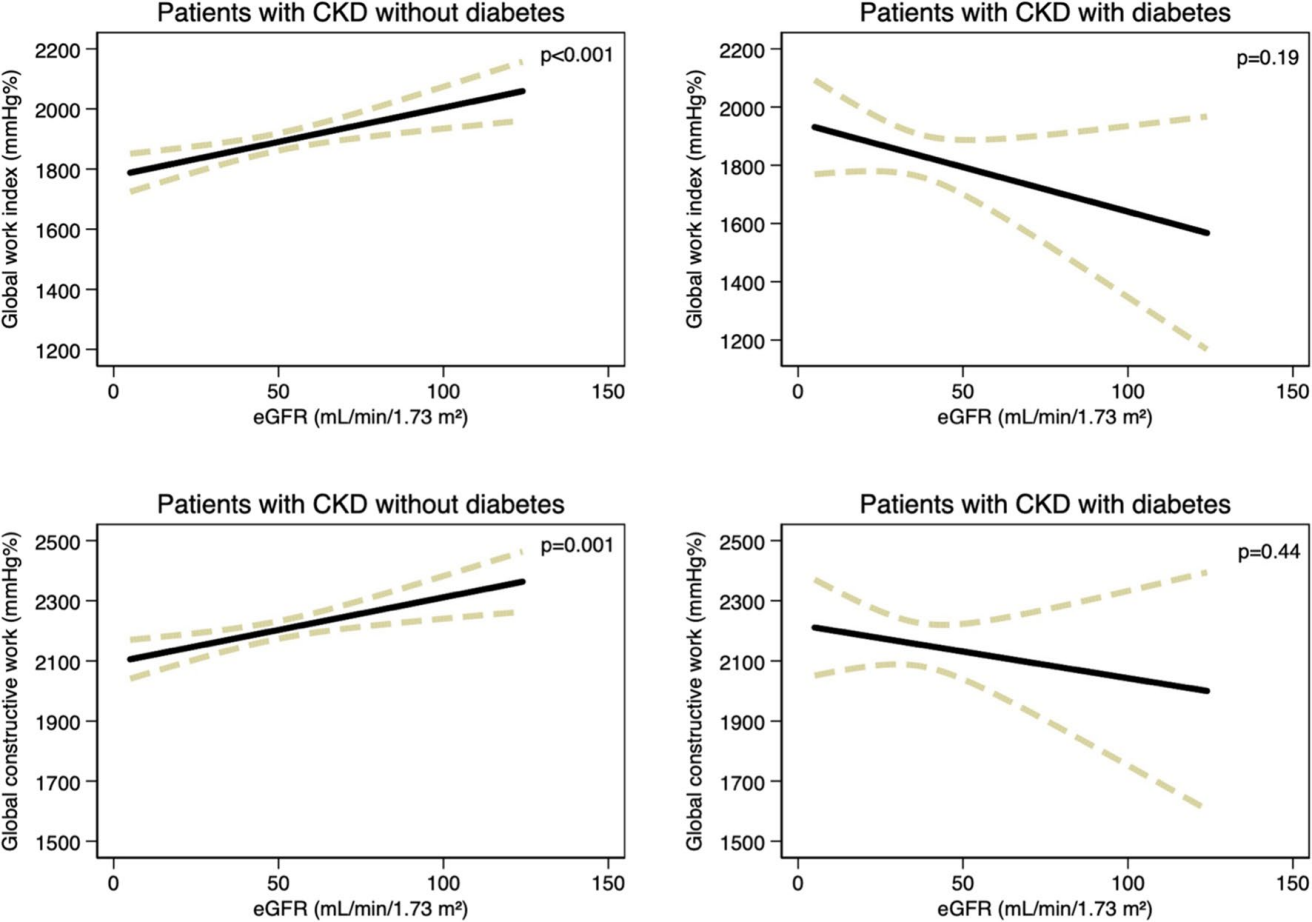
The association between eGFR and the global work index in patients without diabetes (top left panel) and patients with diabetes (top right panel). In addition, the association between eGFR and global constructive work is also depicted in patients without diabetes (bottom left panel) and patients with diabetes (bottom right panel). CKD, chronic kidney disease; eGFR, estimated glomerular filtration rate

No effect modification from hypertension or CKD etiology was identified; however, diabetes did significantly modify the association between eGFR and both GWI and GCW (*p* for interaction = 0.007 and 0.033, respectively) but not for GWW nor GWE (*p* for interaction > 0.05). These effect modifications persisted in multivariable adjustments (*p* for interaction = 0.010 and 0.036 for GWI and GCW, respectively), such that per 10 mL/min/1.73 m^2^ decrease in eGFR, GWI decreased by 22 (9–35) mmHg% (*p* = 0.001) and GCW decreased by 21 (8–34) mmHg% (*p* = 0.001) in patients without diabetes, whereas no significant associations were observed in patients with diabetes (Fig. 3). In a subgroup of patients with current signs of functional kidney disease (*n*, 670), similar observations were made for GWI but not for GCW (Supplementary Results).

Logistic regression revealed that decreasing eGFR was associated with an increased likelihood of abnormalities in all work measures. However, diabetes modified the associations between eGFR and abnormal GWI (*p* for interaction = 0.001), abnormal GWW (*p* for interaction = 0.032), and abnormal GWE (*p* for interaction = 0.002) but not for abnormal GCW (*p* for interaction = 0.32). The associations were modified such that decreasing eGFR was associated with an elevated likelihood of abnormal GWI (OR 1.13 (1.04–1.23), per 10 mL/min/1.73 m^2^ decrease, *p* = 0.005) in patients without diabetes but a lower likelihood in patients with diabetes (Supplemental Fig. 1). Decreasing eGFR was also associated with an elevated likelihood of abnormal GCW (OR 1.14 (1.02–1.29), per 10 mL/min/1.73 m^2^ decrease, *p* = 0.027), GWW (OR 1.21 (1.12–1.29), per 10 mL/min/1.73 m^2^ decrease, *p* < 0.001), and GWE (OR 1.18 (1.10–1.27), per 10 mL/min/1.73 m^2^ decrease, *p* < 0.001) in patients without diabetes whereas no association between eGFR and abnormal GCW, GWW, and GWE were observed in patients with diabetes (*p* > 0.05).

**Fig. 4:**
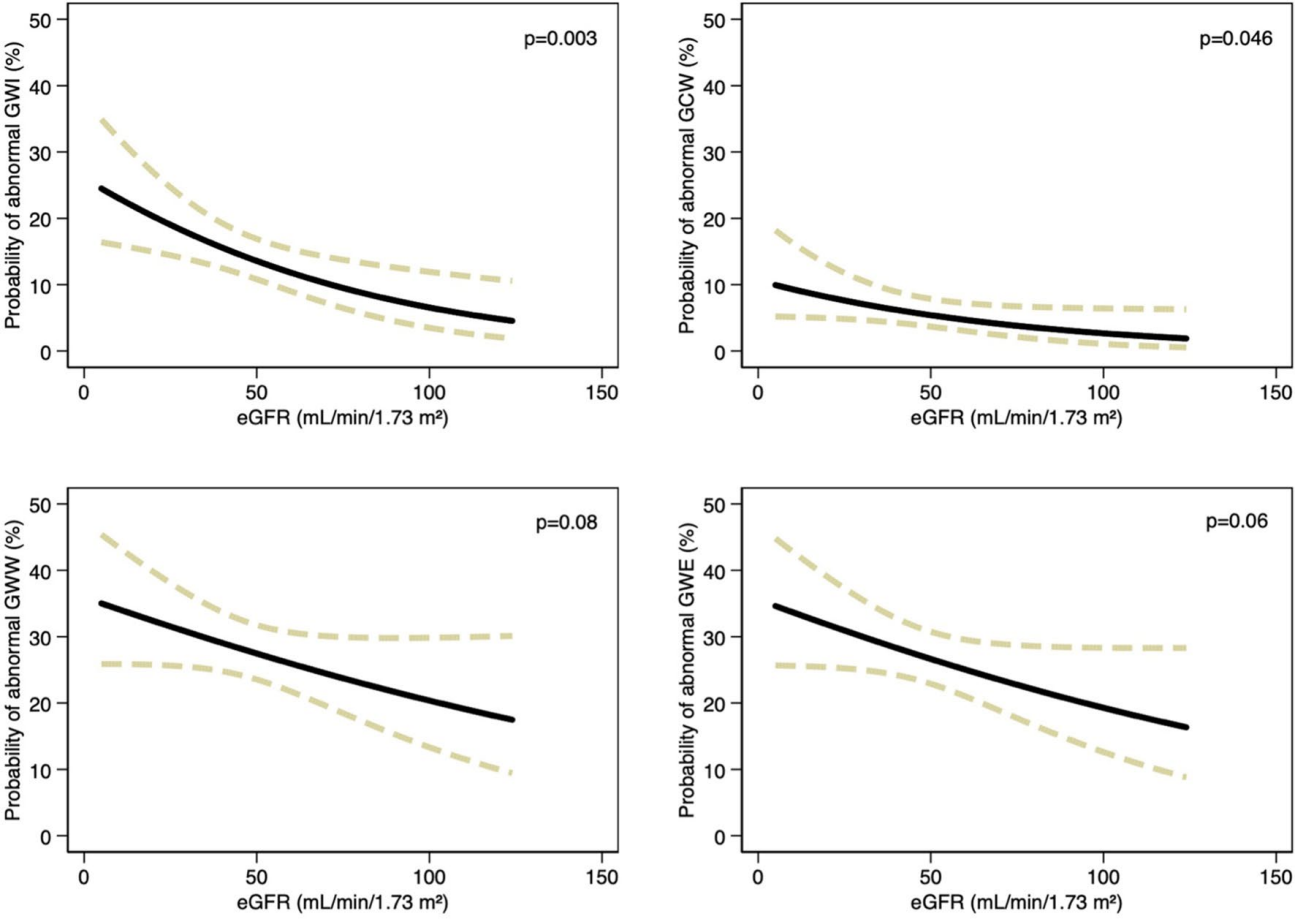
Based on logistic regression, these figures illustrate the association between eGFR and the probability of having abnormal GWI (top left panel), GCW (top right panel), GWW (bottom left panel), and GWE (bottom right panel) after multivariable adjustments in patients without diabetes. eGFR, estimated glomerular filtration rate; GWI, global work index; GCW, global constructive work; GWW, global wasted work; GWE, global work efficiency

These effect modifications persisted in multivariable regression models (abnormal GWI, *p* for interaction = 0.002; abnormal GWW, *p* for interaction = 0.043; abnormal GWE, *p* for interaction = 0.009). After multivariable adjustments, decreasing eGFR remained significantly associated with an increased likelihood of having abnormal GWI and GCW but not GWW nor GWE (Fig. 4), whereas no association between eGFR and abnormal work measures were observed in patients with diabetes (*p* > 0.05 in all analyses).

In logistic regression, we observed that both eGFR groups G3 and G4 + 5 were significantly associated with an increased likelihood of having abnormal GWI, GCW, GWW, and GWE as compared to the G1 + 2 group in patients without diabetes but not in patients with diabetes (Supplementary Table 1). After multivariable adjustment, these associations persisted for abnormal GWI and GCW in the patients without diabetes (Supplementary Table 1).

### Myocardial work in relation to albuminuria

When stratified by degree of albuminuria, we did not observe any differences in myocardial work measures across the categories of normo-, micro-, or macroalbuminuria (Table 3). Similarly, no associations between UACR and work measures were observed in linear regression analyses (Supplementary Fig. 2).

**Table 3:** Myocardial work according to albuminuria

	Normoalbuminuria UACR < 30 mg/g *n* = 228	Microalbuminuria UACR 30–300 mg/g *n* = 232	Macroalbuminuria UACR > 300 mg/g *n* = 273	*p* for trend
Myocardial work measures				
Global work index, mmHg%	1880 ± 393	1866 ± 402	1885 ± 442	0.88
Global constructive work, mmHg%	2184 ± 409	2187 ± 402	2201 ± 457	0.89
Global wasted work, mmHg%	126 [95;182]	133 [96;186]	141 [100;182]	0.18
Global work efficiency, %	94.6 [92.6;95.9]	94.3 [91.9;95.7]	94.2 [92.1;95.9]	0.23
Abnormal work measures				
Abnormal global work index, *n* (%)	37 (16)	49 (21)	52 (19)	0.40
Abnormal global constructive work, *n* (%)	19 (8)	20 (9)	32 (12)	0.36
Abnormal global wasted work, *n* (%)	73 (32)	84 (36)	100 (37)	0.51
Abnormal global work efficiency, *n* (%)	69 (30)	78 (34)	97 (36)	0.46

*UACR*, urine albumin-creatinine ratio

By extension, logistic regression revealed that neither the presence of micro- or macroalbuminuria was associated with an elevated likelihood of having abnormal GWI, GCW, GWW, nor GWE as compared to normoalbuminuria (*p* > 0.05 in all analyses).

Of note, tests for interaction did not reveal any effect modification from hypertension, CKD etiology, or diabetes in either linear or logistic regression analyses.

## Discussion

Based on the largest prospective study on myocardial work in patients with CKD, we observed the following: Patients with CKD exhibited a higher amount of wasted work, resulting in reduced work efficiency, as compared to controls. While no differences were observed in myocardial work (the global work index), the findings were influenced by left ventricular geometry such that myocardial work indeed decreased in patients with LVH. In addition, decreasing eGFR was associated with lower myocardial work in patients without diabetes, and eGFR < 60 mL/min/1.73 m^2^ was associated with increased likelihood of abnormal myocardial work parameters. Finally, albuminuria was not associated with myocardial work measures.

### Myocardial work in CKD

The recent technological advancement allowing for the acquisition of myocardial work indices has provided a promising tool to detect subclinical systolic dysfunction by incorporating the strengths of the robust marker, GLS, while accounting for afterload [19]. Indeed myocardial work parameters have shown potential value beyond GLS in other studies [21], and given the altered afterload conditions often observed with CKD, exploration into measures of ventriculo-arterial coupling is warranted.

A handful of studies have already investigated myocardial work in CKD, primarily by comparing myocardial work in a case–control design (number of patients with CKD ranging from 68 to 144). These have repeatedly shown that patients with CKD have higher wasted work and lower work efficiency compared to controls [22–24], consistent with our findings. Since work efficiency is a derivative of wasted and constructive work, this finding is driven by the higher amount of wasted work. Wasted work represents the amount of work generated that does not translate into cardiac output. Although this can be observed in the presence of electromechanical dyssynchrony [25], our findings remained unchanged after adjusting for presence of left bundle branch block, suggesting other mechanisms behind our finding. Two predominant mechanisms contribute to wasted work, early systolic lengthening and post-systolic shortening [11]. Historically, these features have been considered signs of myocardial ischemia, frequently observed throughout the entire cascade of myocardial ischemia, with their presence indicating an elevated risk of cardiovascular events [26–28]; hence, the finding of a higher amount of wasted work may suggest presence of coronary artery disease, a well-established comorbidity associated with CKD.

Even though no differences were noted in myocardial work, it is interesting to note that several studies — ours included — have suggested that left ventricular geometry influences myocardial work measures in patients with CKD but diverging results have been reported on this.

In 33 patients on chronic hemodialysis with LVH vs. 35 controls, Liu et al. observed lower GWI consistent with our findings [22]. However, another study of patients with CKD (defined as eGFR < 60 mL/min/1.73 m^2^ for over 3 months) that included 46 patients with LVH patients, 59 patients without LVH, and 33 healthy controls revealed higher GWI in those with CKD and LVH than those without LVH and the healthy controls [24]. Several aspects may contribute to these diverging findings. The differences in study populations in particular make it difficult to compare the studies since the duration, degree, and underlying cause of CKD could influence the findings (details that are not available in all related studies). The discrepancy in findings may, however, reflect differences in duration of LVH, since shorter-lasting LVH could indicate that the left ventricle was compensating by performing more work against an increased workload. In our study and in the study on hemodialysis patients [22], the remodeling process may have lasted longer and resulted in hypertrophy with myocyte disarray and interstitial fibrosis precipitating decompensation and worsening systolic function [29]. However, further studies, in particular longitudinal and outcome studies, are needed to substantiate this hypothesis by examining how myocardial work measures develop with changes in left ventricular geometry and whether this translates into worse prognosis, which has been suggested from community-based cohort studies [30].

### Myocardial work in relation to eGFR

A notable finding in our study was that eGFR was associated with GWI and GCW in patients without diabetes, whereas no association between eGFR and any myocardial work measure was observed in patients with diabetes. No other study has previously examined the interplay between eGFR and diabetes in relation to myocardial work and it is unclear why decreasing eGFR would not lead to deterioration in work measures in patients with diabetes, particularly considering that the cardiorenal syndrome in diabetes is well-established [31]. Our findings therefore require external validation and deeper exploration into diabetes characteristics to shed further light on this finding.

Even though the effect modification from diabetes is previously unrecognized, the previously mentioned studies on myocardial work in CKD have explored the potential association between eGFR and myocardial work. Liu et al. investigated clinical correlates to myocardial work measures and reported that eGFR was associated with GWW and GWE [24]. This is consistent with our observations; however, we did not find that these associations were independent of confounders. Whether this also pertains to the findings from Liu et al. is unclear since multivariable analyses were not reported. By contrast, Liu et al. reported independent associations between eGFR and GWW [23]. However, whether GWW was appropriately transformed for regression analysis is unclear. In addition, our study had markedly greater power that allowed for more extensive adjustments. Finally, it is unclear whether a sole finding of higher GWW — that does not translate into lower GWE — is clinically relevant. In fact, only the study by Ke et al. that evaluated 93 patients with CKD observed an association between eGFR and GWI [32], similar to our study. Our findings further extend those findings to suggest that decreasing eGFR below 60 mL/min/1.73 m^2^ may be used to indicate the presence of abnormal myocardial work. However, it is still unclear whether the identification of this abnormality in myocardial work also indicates an elevated likelihood of cardiovascular outcome in patients with CKD. It is also unclear whether treatment with heart failure medication would improve myocardial work in patients with CKD, even though this has been alluded to in a prospective, but non-randomized study of patients with end-stage renal disease [33].

### Myocardial work in relation to albuminuria

Interestingly, we did not find albuminuria to be associated with myocardial work measures, which extends our previous findings also showing a lack of association between both standard echocardiographic measures of cardiac function and GLS to UACR. Possible explanations have been discussed in our previous report [13], but a principal reason for this may be that our study included a heterogeneous sample of patients with CKD, some with etiologies that may not lead to albuminuria. However, no previous studies have examined the relationship between myocardial work and UACR, and further studies are consequently needed to validate our findings.

### Limitations

Since we did not include patients on dialysis, our findings cannot be extrapolated to these patients. Similarly, our findings cannot be applied to patients with aortic valve stenosis nor severe aortic regurgitation as the non-invasive pressure estimation does not apply in this setting. Due to the observational nature of the study and lack of relevant parameters, including NT-proBNP, Cystatin C, and SGLT2-inhibitor usage, our study may be subject to residual and uncorrected confounding. This also pertains to the fact that heart failure may be underestimated in this study, since heart failure with preserved ejection fraction may be an underdiagnosed condition in Denmark [34]. The study was conducted before SGLT2-inhibitors became standard of care in CKD, making our findings less generalizable to contemporary patients with CKD.

Furthermore, since approximately a third of the controls presented with hypertension, we cannot exclude that this could have influenced our findings; however, we sought to account for this through multivariable adjustments.

## Conclusion

Patients with CKD and left ventricular hypertrophy exhibited poorer systolic function by myocardial work compared to matched controls. Furthermore, patients with CKD exhibited lower work efficiency and a higher amount of wasted work.

In patients without diabetes, decreasing eGFR was associated with decreasing myocardial work, which was not the case for patients with diabetes, and eGFR below 60 mL/min/1.73 m^2^ could indicate abnormal myocardial work in patients with CKD without diabetes.

No association between albuminuria and myocardial work was observed in patients with CKD.

## Supplementary Information

Below is the link to the electronic supplementary material.

Supplementary file1 (DOCX 197 KB)

## Data Availability

The data underlying this study contains sensitive patient information and can therefore not be shared publicly according to Danish legislation. Methodology will be shared upon reasonable request.

## References

[1] Eknoyan G, Levey AS, Levin NW, Keane WF (2001) The national epidemic of chronic kidney disease. What we know and what we can do. Postgrad Med 110:23–29 11570203 10.3810/pgm.2001.09.1024

[2] Lv J-C, Zhang L-X (2019) Prevalence and disease burden of chronic kidney disease. Adv Exp Med Biol 1165:3–15 31399958 10.1007/978-981-13-8871-2_1

[3] Zelnick LR, Shlipak MG, Soliman EZ et al (2022) Prediction of incident heart failure in CKD: the CRIC study. Kidney Int Rep 7:708–719 35497796 10.1016/j.ekir.2022.01.1067 PMC9039424

[4] Kottgen A, Russell SD, Loehr LR et al (2007) Reduced kidney function as a risk factor for incident heart failure: the Atherosclerosis Risk in Communities (ARIC) study. J Am Soc Nephrol 18:1307–1315 17344421 10.1681/ASN.2006101159

[5] Bansal N, Zelnick L, Bhat Z et al (2019) Burden and outcomes of heart failure hospitalizations in adults with chronic kidney disease. J Am Coll Cardiol 73:2691–2700 31146814 10.1016/j.jacc.2019.02.071 PMC6590908

[6] Matsushita K, Kwak L, Sang Y et al (2017) Kidney disease measures and left ventricular structure and function: the Atherosclerosis Risk in Communities study. J Am Heart Assoc 6:e006259 28939714 10.1161/JAHA.117.006259 PMC5634280

[7] Cai Q-Z, Lu X-Z, Lu Y, Wang AY-M (2014) Longitudinal changes of cardiac structure and function in CKD (CASCADE study). J Am Soc Nephrol 25:1599–1608 24525033 10.1681/ASN.2013080899 PMC4073437

[8] Park M, Hsu C, Li Y et al (2012) Associations between kidney function and subclinical cardiac abnormalities in CKD. J Am Soc Nephrol 23:1725–1734 22935481 10.1681/ASN.2012020145 PMC3458463

[9] Cikes M, Solomon SD (2016) Beyond ejection fraction: an integrative approach for assessment of cardiac structure and function in heart failure. Eur Heart J 37:1642–1650 26417058 10.1093/eurheartj/ehv510

[10] Russell K, Eriksen M, Aaberge L et al (2012) A novel clinical method for quantification of regional left ventricular pressure-strain loop area: a non-invasive index of myocardial work. Eur Heart J 33:724–733 22315346 10.1093/eurheartj/ehs016 PMC3303715

[11] Boe E, Skulstad H, Smiseth OA (2019) Myocardial work by echocardiography: a novel method ready for clinical testing. Eur Heart J Cardiovasc Imaging 20:18–20 30376059 10.1093/ehjci/jey156

[12] Sørensen IMH, Saurbrey SAK, Hjortkjær HØ et al (2020) Regional distribution and severity of arterial calcification in patients with chronic kidney disease stages 1–5: a cross-sectional study of the Copenhagen chronic kidney disease cohort. BMC Nephrol 21:534 33297991 10.1186/s12882-020-02192-y PMC7726904

[13] Landler NE, Olsen FJ, Christensen J et al (2022) Associations between albuminuria, estimated GFR and cardiac phenotype in a cohort with chronic kidney disease: the CPH-CKD ECHO study. J Card Fail 28:1615–1627 36126901 10.1016/j.cardfail.2022.09.002

[14] Kidney Disease: Improving Global Outcomes (KDIGO) CKD Work Group (2013) KDIGO clinical practice guideline for the evaluation and management of chronic kidney disease. Kidney Int Suppl 3:1–150 10.1016/j.kisu.2017.10.001 PMC6341011 30681074

[15] Levey AS, Stevens LA, Schmid CH et al (2009) A new equation to estimate glomerular filtration rate. Ann Intern Med 150:604–612 19414839 10.7326/0003-4819-150-9-200905050-00006 PMC2763564

[16] Lang RM, Badano LP, Mor-Avi V et al (2015) Recommendations for cardiac chamber quantification by echocardiography in adults: an update from the American Society of Echocardiography and the European Association of Cardiovascular Imaging. Eur Heart J Cardiovasc Imaging 16:233–270 25712077 10.1093/ehjci/jev014

[17] Lancellotti P, Tribouilloy C, Hagendorff A et al (2013) Recommendations for the echocardiographic assessment of native valvular regurgitation: an executive summary from the European Association of Cardiovascular Imaging. Eur Heart J Cardiovasc Imaging 14:611–644 23733442 10.1093/ehjci/jet105

[18] Baumgartner H, Hung J, Bermejo J et al (2009) Echocardiographic assessment of valve stenosis: EAE/ASE recommendations for clinical practice. Eur J Echocardiogr 10:1–25 19065003 10.1093/ejechocard/jen303

[19] Smiseth OA, Donal E, Penicka M, Sletten OJ (2021) How to measure left ventricular myocardial work by pressure-strain loops. Eur Heart J Cardiovasc Imaging 22:259–261 33257982 10.1093/ehjci/jeaa301

[20] Olsen FJ, Skaarup KG, Lassen MCH et al (2022) Normal values for myocardial work indices derived from pressure-strain loop analyses: from the CCHS. Circ Cardiovasc Imaging 15:e013712 35535593 10.1161/CIRCIMAGING.121.013712

[21] Edwards NFA, Scalia GM, Shiino K et al (2019) Global myocardial work is superior to global longitudinal strain to predict significant coronary artery disease in patients with normal left ventricular function and wall motion. J Am Soc Echocardiogr 32:947–957 31043359 10.1016/j.echo.2019.02.014

[22] Liu C, Feng Y-P, Yan Z-N et al (2021) Value of quantitative analysis of left ventricular systolic function in patients on maintenance hemodialysis based on myocardial work technique. BMC Cardiovasc Disord 21:76 33549050 10.1186/s12872-021-01899-6 PMC7866689

[23] Liu F-Z, Wang X-L, Zhang C-Q (2021) Quantitative assessment of left ventricular myocardial work in chronic kidney disease patients by a novel non-invasive pressure-strain loop analysis method. Int J Cardiovasc Imaging 37:1567–1575 33433746 10.1007/s10554-020-02132-9

[24] Liu X, Chen L, Zhong X et al (2022) Noninvasive evaluation of myocardial work in patients with chronic kidney disease using left ventricular pressure-strain loop analysis. Diagnostics (Basel) 12:856 35453914 10.3390/diagnostics12040856 PMC9029752

[25] Vecera J, Penicka M, Eriksen M et al (2016) Wasted septal work in left ventricular dyssynchrony: a novel principle to predict response to cardiac resynchronization therapy. Eur Heart J Cardiovasc Imaging 17:624–632 26921169 10.1093/ehjci/jew019 PMC4871236

[26] Brainin P, Hoffmann S, Fritz-Hansen T et al (2018) Usefulness of postsystolic shortening to diagnose coronary artery disease and predict future cardiovascular events in stable angina pectoris. J Am Soc Echocardiogr 31:870-879.e3 30077186 10.1016/j.echo.2018.05.007

[27] Brainin P, Haahr-Pedersen S, Olsen FJ et al (2020) Early systolic lengthening in patients with ST-segment-elevation myocardial infarction: a novel predictor of cardiovascular events. J Am Heart Assoc 9:e013835 31973603 10.1161/JAHA.119.013835 PMC7033900

[28] Brainin P, Skaarup KG, Iversen AZ et al (2019) Post-systolic shortening predicts heart failure following acute coronary syndrome. Int J Cardiol 276:191–197 30527346 10.1016/j.ijcard.2018.11.106 PMC6365100

[29] Tadic M, Cuspidi C, Marwick TH (2022) Phenotyping the hypertensive heart. Eur Heart J 43:3794–3810 35869979 10.1093/eurheartj/ehac393

[30] Olsen FJ, Skaarup KG, Lassen MCH et al (2024) Association between myocardial work indices and cardiovascular events according to hypertension in the general population. Eur Heart J Cardiovasc Imaging 25:413–424 37930752 10.1093/ehjci/jead292

[31] Méndez Fernández AB, Vergara Arana A, Olivella San Emeterio A et al (2023) Cardiorenal syndrome and diabetes: an evil pairing. Front Cardiovasc Med 10:1185707 37234376 10.3389/fcvm.2023.1185707 PMC10206318

[32] Ke Q-Q, Xu H-B, Bai J et al (2020) Evaluation of global and regional left ventricular myocardial work by echocardiography in patients with chronic kidney disease. Echocardiography 37:1784–1791 33084159 10.1111/echo.14864

[33] Wang B, Wang G-H, Ding X-X et al (2022) Effects of sacubitril/valsartan on resistant hypertension and myocardial work in hemodialysis patients. J Clin Hypertens (Greenwich) 24:300–308 35099841 10.1111/jch.14422 PMC8924992

[34] Madelaire C, Gustafsson F, Køber L et al (2020) Identification of patients with new-onset heart failure and reduced ejection fraction in Danish administrative registers. Clin Epidemiol 12:589–594 32606984 10.2147/CLEP.S251710 PMC7292248

