# Supplementary material for: Myocardial work in chronic kidney disease: insights from the CPH-CKD ECHO Study
Source: Clin Res Cardiol. 2024 May 15;113(11):1576–88. doi: 10.1007/s00392-024-02459-6 (PMC11493787; doi:10.1007/s00392-024-02459-6)
Supplement: Supplementary file 1 — Supplementary file1 (DOCX 197 KB) [file 392_2024_2459_MOESM1_ESM.docx]

**SUPPLEMENTARY MATERIAL**

**Myocardial Work in Chronic Kidney Disease:
Insights from the CPH-CKD ECHO Study**

Flemming Javier Olsen, MD, PhD^1,2^; Nino Emanuel Landler, MD, PhD^1,2^; Jacob Christensen, MB^1,2^;
Bo Feldt-Rasmussen, MD, DMSc^3,4^; Ditte Hansen, MD, PhD^4,5^; Christina Christoffersen, MD, PhD^2,6^; Ellen Linnea Freese Ballegaard, MD^3,4^; Ida Maria Hjelm Sørensen, MD, PhD^3^;
Sasha Saurbrey Bjergfelt, MD^2,3^; Eline Seidelin, MB^5^; Susanne Bro, MD, PhD, DMSc^3^;
Tor Biering-Sørensen, MD, MSc, MPH, PhD^1,2,7,8^

^1^ Department of Cardiology, Copenhagen University Hospital – Herlev and Gentofte, Hellerup, Denmark.

^2^ Department of Biomedical Sciences, University of Copenhagen, Copenhagen, Denmark.

^3^ Department of Nephrology, Copenhagen University Hospital – Rigshospitalet, Copenhagen, Denmark

^4^ Department of Clinical Medicine, University of Copenhagen, Copenhagen, Denmark

^5^ Department of Nephrology, Copenhagen University Hospital - Herlev and Gentofte, Herlev, Denmark

^6^ Department of Clinical Biochemistry, Copenhagen University Hospital – Rigshospitalet, Copenhagen, Denmark
^7^ Department of Cardiology, Copenhagen University Hospital – Rigshospitalet, Copenhagen, Denmark
^8^ Steno Diabetes Center Copenhagen, Copenhagen, Denmark

**Running title:** Myocardial work in kidney disease

**Correspondence:**
Flemming J. Olsen, MD, PhD
Cardiovascular Non-Invasive Imaging Research Laboratory at Dept. of Cardiology, Copenhagen University Hospital - Herlev and Gentofte.
Gentofte Hospitalsvej 8, 2900 Hellerup, Denmark
Phone: +45 31 44 12 29 ; Fax: +45 39 77 73 81
E-mail: [flemming.j.olsen@gmail.com](mailto:flemming.j.olsen@gmail.com)

**Supplementary Results**

In patients with CKD and either reduced eGFR (eGFR<60 mL/min/1.73m^2^) or albuminuria (UACR>30 mg/g), we did not observe any association between eGFR and GWI or GCW (p>0.05 for both), but did observe increasing GWW with decreasing eGFR (p<0.001) and decreasing GWE with decreasing eGFR (p<0.001). However, these associations did not persist after multivariable adjustments (p=0.60 and =0.34, for GWW and GWE, respectively).
However, diabetes significantly modified the association between GWI and eGFR (p for interaction=0.015), but not for any other work measure (p for interaction>0.05 for GCW, GWW, and GWE). This effect modification persisted in multivariable adjustments, such that in patients without diabetes, GWI decreased by 19 mmHg% (5-34 mmHg%) per 10 mL/min/1.73m^2^ decrease in eGFR (p=0.009), whereas no association between GWI and eGFR was observed in patients with diabetes (p=0.20).
No association was observed between UACR and any work measure in this subgroup (p>0.05 for all), and no effect modification from diabetes was observed for the association between UACR and any work measure (p for interaction >0.05 for all).

**Supplemental figure legends**

**Supplemental figure 1**

**Title:** eGFR and abnormal GWI **Caption:** The figure depicts the unadjusted association between eGFR and the likelihood of having abnormal GWI (defined as below 1576 mmHg%). As shown, the association was modified by diabetes such that decreasing eGFR was associated with an increased probability of having abnormal GWI in patients without diabetes, whereas decreasing eGFR was associated with a decreased likelihood of abnormal GWI in patients with diabetes.

eGFR: estimated glomerular filtration rate; GWI: global work index

**Supplemental figure 2**

**Title:** UACR and work measures **Caption:** The figure shows the unadjusted continuous association between UACR and GWI (top left panel), GCW (top right panel), GWW (bottom left panel), and GWE (bottom right panel).

Of note, the x-axis is on a logarithmic scale and the values consequently represent geometric means after back-transformation of logarithmic values. Similarly, global wasted work and global work efficiency also represent geometric means after back-transformation from logarithmic scales.
UACR: urine albumin-creatinine ratio; GWI: global work index; GCW: global constructive work; GWW: global wasted work; GWE: global work efficiency

**Supplementary Table 1 – Association between eGFR and abnormal work**

| Patients without diabetes n: 614 | | | | |
| --- | --- | --- | --- | --- |
|  | Unadjusted OR (95% CI) | Unadjusted P-value | Adjusted OR (95% CI) | Adjusted P-value |
| Association to abnormal GWI (104 events) | | | | |
| G1+2 (eGFR ≥ 60) | Ref. | Ref. | Ref. | Ref. |
| G3 (eGFR 30-59) | 2.75 (1.52-4.98) | 0.001 | 4.22 (2.09-8.53) | <0.001 |
| G4+5 (eGFR ≤ 29) | 3.24 (1.71-6.14) | <0.001 | 3.87 (1.73-8.65) | 0.001 |
| Association to abnormal GCW (53 events) | | | | |
| G1+2 (eGFR ≥ 60) | Ref. | Ref. | Ref. | Ref. |
| G3 (eGFR 30-59) | 2.45 (1.09-5.54) | 0.031 | 3.85 (1.45-10.18) | 0.007 |
| G4+5 (eGFR ≤ 29) | 3.35 (1.43-7.89) | 0.006 | 3.20 (1.07-9.60) | 0.038 |
| Association to abnormal GWW (195 events) | | | | |
| G1+2 (eGFR ≥ 60) | Ref. | Ref. | Ref. | Ref. |
| G3 (eGFR 30-59) | 2.10 (1.36-3.23) | 0.001 | 1.25 (0.76-2.06) | 0.38 |
| G4+5 (eGFR ≤ 29) | 2.78 (1.72-4.49) | <0.001 | 1.41 (0.78-2.52) | 0.25 |
| Association to abnormal GWE (184 events) | | | | |
| G1+2 (eGFR ≥ 60) | Ref. | Ref. | Ref. | Ref. |
| G3 (eGFR 30-59) | 2.44 (1.56-3.82) | <0.001 | 1.65 (1.00-2.74) | 0.051 |
| G4+5 (eGFR ≤ 29) | 2.87 (1.75-4.71) | <0.001 | 1.65 (0.92-2.98) | 0.09 |

eGFR: estimated glomerular filtration rate; GWI: global work index; GCW: global constructive work; GWW: global wasted work; GWE: global work efficiency

| Patients with diabetes  n: 143 | | | | |
| --- | --- | --- | --- | --- |
|  | Unadjusted OR (95% CI) | Unadjusted P-value | Adjusted OR (95% CI) | Adjusted P-value |
| Association to abnormal GWI (40 events) | | | | |
| G1+2 (eGFR ≥ 60) | Ref. | Ref. | Ref. | Ref. |
| G3 (eGFR 30-59) | 0.42 (0.11-1.59) | 0.20 | 0.55 (0.11-2.73) | 0.47 |
| G4+5 (eGFR ≤ 29) | 0.26 (0.06-1.06) | 0.06 | 0.42 (0.07-2.38) | 0.33 |
| Association to abnormal GCW (22 events) | | | | |
| G1+2 (eGFR ≥ 60) | Ref. | Ref. | Ref. | Ref. |
| G3 (eGFR 30-59) | 1.80 (0.21-15.36) | 0.59 | 0.99 (0.08-12.27) | 0.99 |
| G4+5 (eGFR ≤ 29) | 1.50 (0.16-13.75) | 0.72 | 1.51 (0.11-21.49) | 0.76 |
| Association to abnormal GWW (70 events) | | | | |
| G1+2 (eGFR ≥ 60) | Ref. | Ref. | Ref. | Ref. |
| G3 (eGFR 30-59) | 0.61 (0.16-2.30) | 0.46 | 0.27 (0.06-1.28) | 0.10 |
| G4+5 (eGFR ≤ 29) | 0.64 (0.16-2.55) | 0.53 | 0.20 (0.04-1.08) | 0.06 |
| Association to abnormal GWE (67 events) | | | | |
| G1+2 (eGFR ≥ 60) | Ref. | Ref. | Ref. | Ref. |
| G3 (eGFR 30-59) | 0.39 (0.09-1.61) | 0.19 | 0.27 (0.05-1.32) | 0.11 |
| G4+5 (eGFR ≤ 29) | 0.30 (0.07-1.28) | 0.10 | 0.16 (0.03-0.92) | 0.040 |

eGFR: estimated glomerular filtration rate; GWI: global work index; GCW: global constructive work; GWW: global wasted work; GWE: global work efficiency

**Supplementary Figure 1**


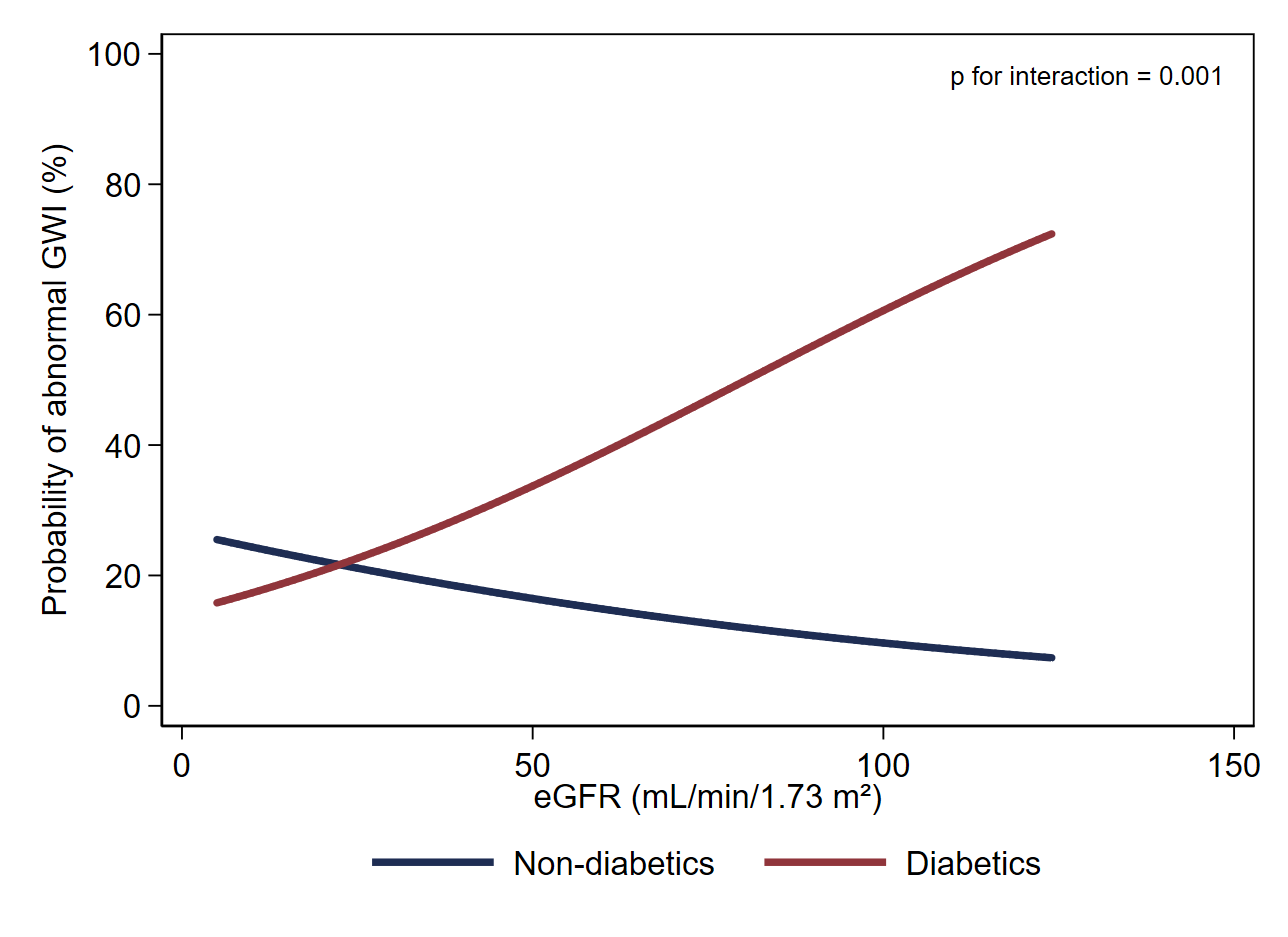


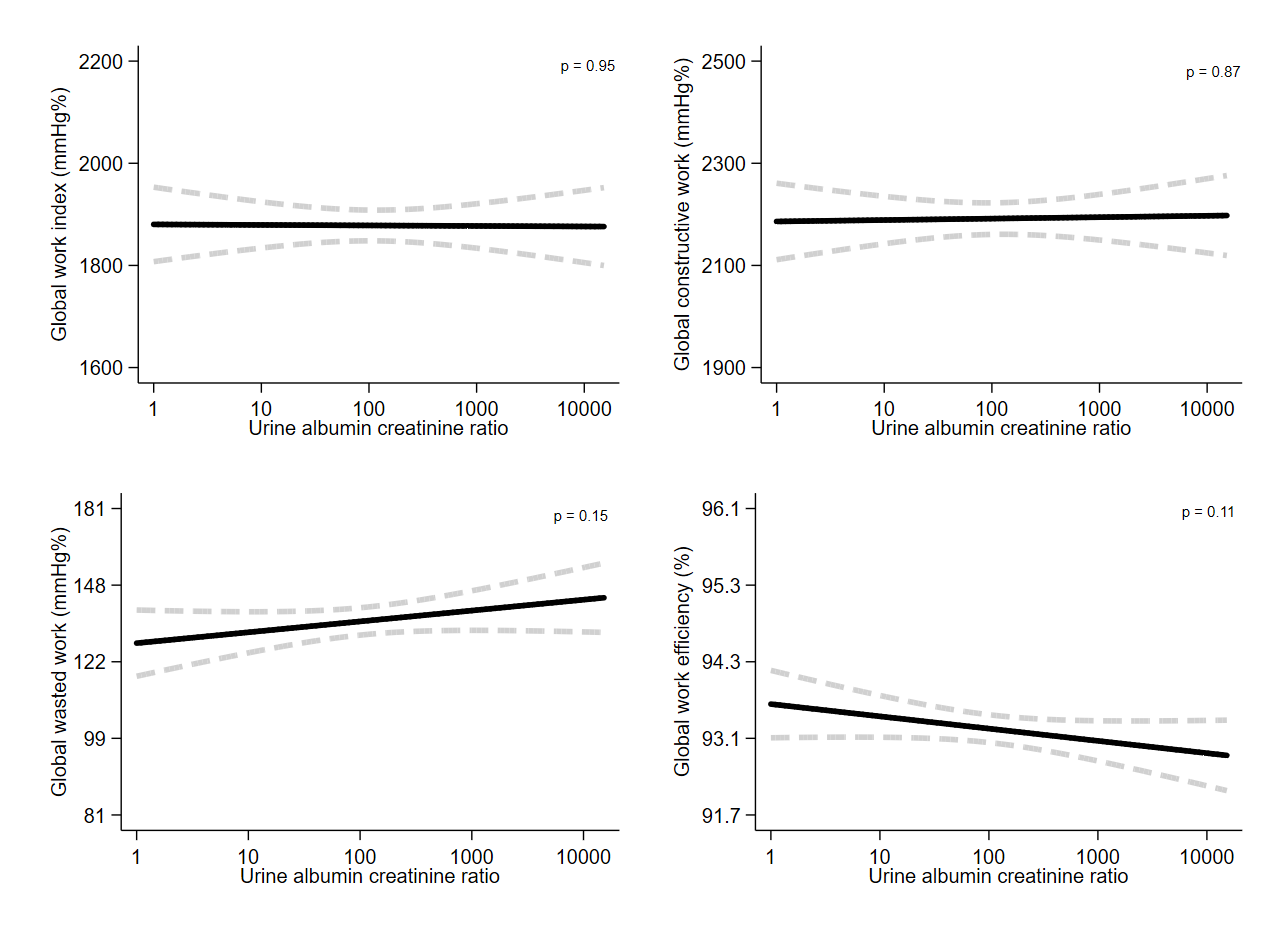
**Supplementary Figure 2**
